# Genetic dissection of drought tolerance in chickpea (*Cicer* *arietinum* L.)

**DOI:** 10.1007/s00122-013-2230-6

**Published:** 2013-12-11

**Authors:** Rajeev K. Varshney, Mahendar Thudi, Spurthi N. Nayak, Pooran M. Gaur, Junichi Kashiwagi, Lakshmanan Krishnamurthy, Deepa Jaganathan, Jahnavi Koppolu, Abhishek Bohra, Shailesh Tripathi, Abhishek Rathore, Aravind K. Jukanti, Veera Jayalakshmi, Anilkumar Vemula, S. J. Singh, Mohammad Yasin, M. S. Sheshshayee, K. P. Viswanatha

**Affiliations:** 1 International Crops Research Institute for the Semi-Arid Tropics (ICRISAT), Hyderabad, India; 2 CGIAR Generation Challenge Programme, c/o CIMMYT, Mexico, DF Mexico; 3 Present Address: University of Florida, Florida, USA; 4 Hokkaido University, Sapporo, Japan; 5 Present Address: Leibniz Institute of Plant Genetics and Crop Plant Research (IPK), Gatersleben, Germany; 6 Present Address: Indian Institute of Pulses Research (IIPR), Kanpur, India; 7 Present Address: Indian Agricultural Research Institute (IARI), New Delhi, India; 8 Present Address: Central Arid Zone Research Institute (CAZRI), Jodhpur, India; 9 ANGRAU Regional Agricultural Research Station, Nandyal, India; 10 RAU-Agricultural Research Station, Durgapura, India; 11 RAK College of Agriculture, Sehore, India; 12 University of Agricultural Sciences - Bangalore, Bangalore, India

## Abstract

*****Key message***:**

**Analysis of phenotypic data for 20 drought tolerance traits in 1–7 seasons at 1–5 locations together with genetic mapping data for two mapping populations provided 9 QTL clusters of which one present on CaLG04 has a high potential to enhance drought tolerance in chickpea improvement.**

**Abstract:**

Chickpea (*Cicer arietinum* L.) is the second most important grain legume cultivated by resource poor farmers in the arid and semi-arid regions of the world. Drought is one of the major constraints leading up to 50 % production losses in chickpea. In order to dissect the complex nature of drought tolerance and to use genomics tools for enhancing yield of chickpea under drought conditions, two mapping populations—ICCRIL03 (ICC 4958 × ICC 1882) and ICCRIL04 (ICC 283 × ICC 8261) segregating for drought tolerance-related root traits were phenotyped for a total of 20 drought component traits in 1–7 seasons at 1–5 locations in India. Individual genetic maps comprising 241 loci and 168 loci for ICCRIL03 and ICCRIL04, respectively, and a consensus genetic map comprising 352 loci were constructed (http://cmap.icrisat.ac.in/cmap/sm/cp/varshney/). Analysis of extensive genotypic and precise phenotypic data revealed 45 robust main-effect QTLs (M-QTLs) explaining up to 58.20 % phenotypic variation and 973 epistatic QTLs (E-QTLs) explaining up to 92.19 % phenotypic variation for several target traits. Nine QTL clusters containing QTLs for several drought tolerance traits have been identified that can be targeted for molecular breeding. Among these clusters, one cluster harboring 48 % robust M-QTLs for 12 traits and explaining about 58.20 % phenotypic variation present on CaLG04 has been referred as “*QTL-hotspot*”. This genomic region contains seven SSR markers (ICCM0249, NCPGR127, TAA170, NCPGR21, TR11, GA24 and STMS11). Introgression of this region into elite cultivars is expected to enhance drought tolerance in chickpea.

**Electronic supplementary material:**

The online version of this article (doi:10.1007/s00122-013-2230-6) contains supplementary material, which is available to authorized users.

## Introduction

Climate change is a global phenomenon that has started to have adverse impact on agriculture. The global temperature is predicted to rise by 2.5 to 4.3 °C by the end of the century (IPCC 2007). The situation is further likely to be exacerbated by the occurrence of increase in the irregularity of rainfall, drought, flood and land degradation. Higher temperatures, more hot days and heat waves are very likely to hit over nearly all land areas. In this context, drought remains as a big challenge while addressing the problem of food insecurity, hunger and malnutrition especially in the areas where people mainly depend on subsistence farming as a major source of their livelihood (Tuberosa 2012).

Chickpea (*Cicer arietinum* L.) is grown on low input marginal lands and represents an important component of the subsistence farming. It is the second most important grain legume globally cultivated on an area of 13.20 million hectare (Mha) with an annual production of 11.62 million tons (Mt; FAOSTAT 2011). The global demand for chickpea in 2020 is projected to be 17.0 Mt (up from the current 8.6 Mt; Abate et al. 2012). It is mostly grown on residual moisture from monsoon rains on the Indian subcontinent and semi-arid regions of Sub-Saharan Africa (SSA). India is the largest producer and consumer of chickpea. Among various kinds of abiotic (salinity, heat) stresses affecting the chickpea production, drought stress particularly at the end of the growing season is a major constraint to chickpea production and yield stability in arid and semi-arid regions of the world (see Krishnamurthy et al. 2010). Drought causes substantial annual yield losses up to 50 % in chickpea and the productivity remained constant for the past six decades (Ahmad et al. 2005; see Varshney et al. 2010). With predicted climate change scenarios and continuous population explosion, there is a great need to develop high-yielding chickpea varieties with improved drought tolerance (Krishnamurthy et al. 2013a).

Drought tolerance is a generic term for a highly complex phenomenon of plant responses. In a practical sense, it is the relative ability of the crop to sustain adequate biomass production and maximize crop yield under increasing water deficit throughout the growing season, rather than the physiological aptitude of the plant for its survival (Serraj and Sinclair 2002). In such context, tolerance to drought is a complex trait with quantitative nature and the underlying mechanism may be due to drought escape, avoidance and tolerance in many crops. Chickpea yields are highly prone to large genotype by environment (*G* × *E*) interactions in marginal environments (Kashiwagi et al. 2008). Breeding for yield under drought conditions using conventional approaches has not been quite successful over the years due to this instability and the poor heritability. Under such circumstances, molecular breeding seems to be a better strategy that can be deployed by targeting drought tolerance component traits with the help of molecular markers.

Understanding genetic basis and identification of molecular markers for drought tolerance component traits are prerequisites for deploying molecular breeding for developing superior genotypes of chickpea. Very recently, significant progress has been made in developing molecular markers and genetic maps in chickpea (Nayak et al. 2010; Gujaria et al. 2011; Gaur et al. 2011; Thudi et al. 2011; Hiremath et al. 2012). While several mapping studies have targeted biotic stress tolerance loci (see Millàn et al. 2006), drought tolerance trait has not yet been targeted systematically for molecular mapping in chickpea. Precision of molecular mapping of a trait, however, is a direct function of precise phenotyping of the trait (Tuberosa 2012; Mir et al. 2012). In the context of drought tolerance, the structure and function of the root system is expected to directly contribute to the transpiration while that of the shoot system structure and function to the transpiration efficiency (TE). Despite their importance in drought tolerance, the roots have attracted little attention in genetic studies mainly because of hard work and skills required for phenotyping root traits (Varshney et al. 2011). As a result of hard work for several years, semi-automated and high-throughput phenotyping techniques for root traits were established at ICRISAT to assess the genetic variability for the root traits in the germplasm collection of chickpea (Kashiwagi et al. 2007). As a result of such endeavors, root traits such as root depth, root biomass and root length density (RLD) were identified as most promising traits in chickpea for terminal drought tolerance, as these help in greater extraction of soil moisture (Kashiwagi et al. 2006; Varshney et al. 2011). Importance of such root traits contributing to drought tolerance has also been demonstrated in some other legumes (Wang et al. 2004) and cereals (Toorchi et al. 2004; Tuberosa and Salvi 2007).

In addition to the root traits, another important trait for drought tolerance is water-use efficiency (WUE) or TE (Passioura 1977; Kashiwagi et al. 2013). Carbon isotope discrimination (δ^13^C) is considered the best method to screen germplasm for WUE. While a range of reports are available on correlation between δ^13^C and TE, a positive correlation was found between δ^13^C and TE under drought stress environments in chickpea (Kashiwagi et al. 2006). Furthermore, irrespective of root traits or TE, yield and yield component traits and harvest index (HI) are always considered the most reliable traits for breeding for drought tolerance.

With an objective to dissect drought tolerance into component traits and understand genetic basis and identify molecular markers for different component traits, this study undertakes extensive phenotyping and genotyping and their comprehensive analysis on two intra-specific recombinant inbred line (RIL) populations. This study is the first report on the development of the most-dense genetic maps on intra-specific populations and identification of both main-effect QTLs (M-QTLs) as well as epistatic QTLs (E-QTLs) for different drought tolerance traits in chickpea. Most importantly, this study reports a “*QTL-hotspot*” in the chickpea genome, identified in analysis on both RIL populations, that contain 45 M-QTLs and 973 E-QTLs for several drought tolerance traits contributing up to 58.20 % phenotypic variation for targeted traits. In summary, this study provides molecular markers for deploying molecular breeding for drought tolerance, a very complex trait, to develop superior chickpea varieties.

## Materials and methods

### Plant material and DNA extraction

Based on screening of mini-core collection for drought tolerance-related root traits, ICC 4958 (a drought tolerant breeding line developed by Jawaharlal Nehru Krishi Vishwa Vidyalaya, Jabalpur, Madhya Pradesh, India) and ICC 8261 (a drought tolerant landrace from Lebanon) assembled in ICRISAT’s genebank in 1973 and 1974 were found to possess larger root system, while ICC 283 and ICC 1882 are landraces collected from India and assembled in ICRISAT’s genebank in 1974 and 1973, respectively, were found to possess shorter root system. These phenotypically and genetically distinct genotypes were used for developing two intra-specific mapping populations, namely ICCRIL03 (264 RILs from ICC 4958 × ICC 1882) and ICCRIL04 (288 RILs from ICC 283 × ICC 8261), at ICRISAT.

DNA from parental genotypes as well as from 232 and 234 RILs of ICCRIL03 and ICCRIL04, respectively, was isolated employing high-throughput mini-DNA extraction method as mentioned in Cuc et al. (2008).

### Phenotypic evaluation

The above-mentioned populations (ICCRIL03 comprising 264 RILs and ICCRIL04 comprising 288 RILs) were evaluated for a total of 20 drought tolerance traits including 6 root traits, 6 yield and yield-related traits, 5 morphological traits, 2 phenological traits and 1 physiological trait in three replications in 1–7 seasons (2005–2006, 2006–2007, 2007–2008, 2008–2009, 2009–2010, 2010–2011 and 2011–2012) at 1–5 locations in India, namely Patancheru (PAT), Nandyal (NDL), Durgapura (DUG), Hiriyur (HIR) and Sehore (SEH) (ESM Table S1).

#### Root trait phenotyping under rainout shelter (ROS) conditions

Both populations were phenotyped for root traits such as root length (RL, cm), root length density (RLD, cm cm^−3^), root dry weight (RDW, g), rooting depth (RDp, cm), root surface area (RSA, cm^2^), root volume (RV, cm^3^), ratio between RDW and total plant dry weight (RTR, %), and one morphological trait, shoot dry weight (SDW, g) in cylinder culture in three replications in rainout shelter using semi-automated high-throughput precise phenotyping facility at ICRISAT, Patancheru as described earlier (Kashiwagi et al. 2006). The ICCRIL03 was phenotyped during post-rainy season of 2005 and 2007, while ICCRIL04 was phenotyped during post-rainy season of 2006 and 2010.

#### Morphological, phenological and yield-related traits under field conditions

ICCRIL03 and ICCRIL04 were phenotyped for five morphological traits (plant height, PHT, cm; plant stand, PS; plant width, PWD, cm; primary branches, PBS; secondary branches, SBS), two phenological traits (days to 50 % flowering, DF; days to maturity, DM) and six yield-related traits (100-seed weight, 100-SDW, g; pods per plant, POD; seeds per pod, SPD; Yield, YLD, g; biomass, BM, g; harvest index, HI, %) during post-rainy 2005–2006, 2006–2007, and 2007–2008 seasons under rainfed (RF) environments at PAT. In addition, ICCRIL03 was also phenotyped under rainfed condition during post rainy 2008 at PAT, SEH, DUG and NDL (ESM Table S1).

Furthermore, both populations were phenotyped for the above-mentioned morphological, phenological and yield-related traits under RF and irrigated (IR) environments. ICCRIL03 was phenotyped during post-rainy 2009–2010 at five locations (PAT, NDL, DUG, HIR and SEH), while ICCRIL04 was phenotyped during post-rainy 2010–2011 and 2011–2012 at four locations (PAT, NDL, DUG and SEH).

#### Phenotyping for physiological trait

Delta carbon ratio (δ^13^C) is considered as an indirect measure of TE, which is an important measure of drought tolerance. For estimating the δ^13^C, fourth and fifth fully expanded leaves from top of the stems of ICCRIL03 population were collected during post-rainy season 2008–2009 at four locations PAT, DUG, NDL and SEH as mentioned in Kashiwagi et al. (2006).

#### Analysis of variance, correlations and heritability

The analysis of variance (ANOVA) for all traits was computed considering genotypes as random effect. Best Linear Unbiased Predictors (BLUPs) were estimated by using SAS MIXED procedure (SAS Inst. 2002–2008, SAS V9.2). In addition, the least square means (LSM; genotype as fixed effect), standard error of differences (SED), least significant difference (LSD) and descriptive statistics such as coefficient of determination (*R* ^2^), coefficient of variation (CV) and grand mean were determined for all the traits studied. Genotypic and phenotypic variance components were also estimated to calculate broad sense heritability (*H* ^2^). Drought tolerance index (DTI) and drought susceptibility index (DSI) were computed as mentioned in Golabadi et al. (2006).

### PCR and marker analysis

A total of 2,717 markers including 2,410 simple sequence repeats (SSRs) (311 SSRs from Nayak et al. 2010; 1,344 SSRs from Thudi et al. 2011; 241 SSRs from Winter et al. 1999; 233 SSRs from Lichtenzveig et al. 2005; 181 SSRs from Gaur et al. 2011; 100 SSRs from Sethy et al. 2006), 230 genic molecular markers (GMMs) from Gujaria et al. (2011) and 77 EST-SSRs from Varshney et al. (2009) were screened on the parental lines of two mapping populations.

Polymorphic markers (321 for ICCRIL03 and 230 for ICCRIL04) were used for genotyping respective mapping populations (ESM Table S2). PCR analysis for all SSR markers were performed in 5 μl reaction volume employing GeneAmp^®^ PCR System 9700 DNA thermal cycler (Applied Biosystems, USA). Marker genotyping for ICCM and CaM series SSRs on RILs was done as mentioned in our earlier studies (Nayak et al. 2010; Thudi et al. 2011). Similarly, genotyping for GMMs and diversity arrays technology (DArT) loci was done on the RILs in the same way as mentioned in our earlier studies (Gujaria et al. 2011; Thudi et al. 2011).

### Construction of genetic maps and consensus maps

Genotyping data were assembled for all segregating makers (ESM Table S2) on 232 and 234 RILs of ICCRIL03 and ICCRIL04 mapping populations, respectively, and linkage-based mapping was performed using JoinMap version 4.0 (Van Ooijen 2006) as described in Bohra et al. (2012). A consensus genetic map was derived from two intra-specific mapping populations using software JoinMap 4.0 as described in Bohra et al. (2012).

### QTL analysis

Candidate QTL regions for drought tolerance were identified using two trait mapping approaches: (1) interval mapping for identifying M-QTLs and (2) epistatic interaction analysis for detecting QTL interactions. Composite interval mapping (CIM) was employed for detection of M-QTLs using Windows QTL Cartographer version 2.5 (Wang et al. 2010). In parallel, for detection of E-QTLs, a two-locus QTL analysis or two-dimensional (2D) genome scanning was conducted using software QTLNetwork version 2.0 (http://ibi.zju.edu.cn/software/qtlnetwork/) allowing simultaneous detection of M-QTLs, E-QTLs, and the QTLs involved in epistatic (*Q* × *Q*) and QTL by environment (*Q* × *E*) interactions as described in Gautami et al. (2012). The threshold for declaring QTL is set to *P* value of 0.05 by permutation method (1,000 permutations).

## Results

### Phenotypic trait variation and heritability

The two intra-specific mapping populations ICCRIL03 and ICCRIL04 were phenotyped for a total of 20 drought component traits in 1–7 seasons at 1–5 locations in India. The component traits, their codes and units of measurement, locations, seasons and environments have been listed in Table 1. In addition, DTI and DSI were computed based on phenotypic data from both RF and IR environments. The key features of extensive phenotyping data are given below and detailed analysis such as mean performance, range of trait values, and *H* ^2^ of traits at different locations, environments and seasons on both RILs are provided in ESM Tables S3 and S4.

**Table 1:** Traits, trait codes, units, locations of phenotyping and environments and mapping populations

Names	Code (units)	Names	Code (units)
Root traits		Drought indices	
Root length density	RLD (cm cm^−3^)	Drought tolerance index	DTI
Root dry weight	RDW (g)	Drought susceptibility index	DSI
Rooting depth	RDp (cm)	Locations	
Root surface area	RSA (cm^2^)	Patancheru	PAT
Root volume	RV (cm^3^)	Nandyal	NDL
Root dry weight/total plant dry weight ratio	RTR (%)	Sehore	SEH
Morphological traits		Durgapura	DUG
Shoot dry weight	SDW (g)	Hiriyur	HIR
Plant height	PHT (cm)	Environments	
Plant width	PWD (cm)	Rainfed	RF
Primary branches	PBS	Irrigated	IR
Secondary branches	SBS	Cylinder culture	CC
Phenological traits		Seasons	
Days to 50 % flowering	DF	2005–06	2005
Days to maturity	DM	2006–07	2006
Yield and yield-related traits		2007–08	2007
Pods/plant	POD	2008–09	2008
Seeds/pod	SPD	2009–10	2009
100-seed weight	100SDW (g)	2010–11	2010
Biomass	BM (g)	2011–12	2011
Harvest index	HI (%)	Mapping populations	
Yield	YLD (g)	ICC 4958 × ICC 1882	ICCRIL03
Transpiration efficiency related traits		ICC 283 × ICC 8261	ICCRIL04
Delta carbon ratio	δ^13^C		

#### Root traits

As roots are the first part of the plants exposed to drought stress, six root traits, namely RLD, RDW, RDp, RV, RSA, and RTR, were used for phenotyping of two RIL populations. In ICCRIL03, the genetic variability for RLD among RILs was high in 2007 (0.1–0.47 cm cm^−3^) compared to 2005 (0.22–0.46 cm cm^−3^) at 35 days after sowing (DAS) (ESM Table S3). However, *H* ^2^ was high in 2005 (0.61) compared to 2007 (0.34). The variation among RILs for RDW was high in 2005 (0.43–1.18 g) compared to 2007 (0.27–0.89 g); however, the *H* ^2^ was low in both seasons (0.29 in 2005 and 0.21 in 2007). Although genotypic variability for RDp was observed among RILs in both the seasons, no significant difference was observed between parents of each of two mapping populations (ESM Table S3). Further, the *H* ^2^ was very low for RDp compared to any other root traits studied. The genotypic variability among RILs for RV and RSA was significant in 2005 and non-significant in 2007. The variation for RTR was high in 2007 (21.92–50.9 %) compared to 2005 (22.43–39.23 %); however, *H* ^2^ was high in 2005 (0.56) compared to 2007 (0.26).

In the case of ICCRIL04, at 35 DAS, RLD ranged from 0.20 to 0.45 cm cm^−3^ (2006) and 0.18 to 0.46 cm cm^−3^ (2010). However, the genotypic variability was significant only in 2010 (ESM Table S4). The genotypic variability was highly significant (<0.001) for RDW, RV and RTR in both seasons; however, the *H* ^2^ was moderate for these traits (0.33–0.48).

#### Morphological traits

Drought stress affects several morphological traits and therefore two RIL populations were also phenotyped for SDW, PHT, PWD, PBS and SBS.

In the case of ICCRIL03, at 35 DAS, genetic variability for SDW among RILs was high in 2007 (0.53–2.23 g) compared to 2005 (1.11–2.75 g). Further, significant differences (*P* < 0.0001) for SDW among RILs were observed in both seasons in addition to high *H* ^2^. PHT ranged from 21.6 to 62.4 cm under RF environment in 2008 across four locations PAT, NDL, DUG and SEH (ESM Table S3). Further, genetic variability for PHT was highly significant (*P* < 0.001) under RF environment at PAT, SEH, NDL, and DUG in 2008 with high *H* ^2^ (0.75–0.99). In addition, PHT also differed significantly among RILs in 2009 both at PAT and NDL under RF and IR environments. However, no significant genetic variability for PHT was observed in 2009 under RF and IR environments in case of SEH and DUG (ESM Table S3). In ICCRIL04, at 35 DAS, genetic variability for SDW among RILs was high in 2010 (0.75–2.78 g) compared to 2006 (0.56–2.10 g). Significant difference for PHT was observed at all locations and all environments in 2010 and 2011 except DUG under RF environment in 2010 (ESM Table S4).

#### Phenological traits

Two phenological traits, namely DF and DM, that are important for breeding were recorded on two RIL populations.

In the case of ICCRIL03, phenotyping of these traits showed significant genetic variability for DF under RF environment in 2008 at PAT, SEH and NDL. However, DF did not differ significantly in both RF and IR environments in SEH in 2009. Similarly, in the case of DUG, no significant difference among RILs was noted in IR environment in 2009 (ESM Table S3). For DM, a significant difference among RILs was observed under RF in 2008 at all four locations (PAT, DUG, NDL and SEH) studied. Further, significant differences were also observed for DM under RF and IR environments at PAT, NDL and DUG in 2009. However, in 2009 at SEH under IR environment, there was no significant difference among RILs (ESM Table S3).

In the case of ICCRIL04, DF was significant across all environments and at all locations (PAT, DUG, NDL and SEH) in 2010 in both RF and IR environments. In addition, DF was also significant across five locations (PAT, DUG, NDL, HIR and SEH) under both IR and RF in 2011. Similarly, DM was also significant across all environments (RF and IR) in 2010 and 2011 at all but one location PAT under RF in 2010 (ESM Table S4).

#### Yield and yield-related traits

Yield, especially under drought stress, is the ultimate requirement for farmers and breeders. Therefore, two RIL populations were phenotyped for yield and yield-related traits like POD, SPD, BM, 100SDW, HI and YLD under RF and IR conditions. Among yield-related traits in ICCRIL03, no significant variability was observed for POD and SPD except for PAT in 2009 under IR environment. Except three locations (SEH in 2009 under both environments, PAT in 2009 under RF and DUG in 2008 under RF environment), the *H* ^2^ was high in case of 100SDW at all locations in 2008 and 2009. It (*H* ^2^ value) ranged from 0.64 to 0.99 across locations and environments studied (ESM Table S3). Significant genetic variation for BM was observed among RILs in 2008 under RF at PAT, SEH and NDL. However, in 2009 significant genetic variability for BM was observed only in the case of NDL under both RF and IR environments. In 2008, under RF environment, genetic variability for HI was significant only at PAT and NDL locations with *H* ^2^ of 0.45 and 0.68, respectively. Further in the case of 2009, genetic variability for HI under RF and IR was significantly high in two locations, PAT and NDL.

In the case of ICCRIL04, the genetic variability for 100SDW, BM, YLD and HI was significant among RILs at all locations (PAT, DUG, NDL, HIR and SEH), both seasons (2010 and 2011) and both environments (IR and RF) except BM at PAT in 2011 under RF condition (ESM Table S4). *H* ^2^ for 100SDW across locations and environments ranged from 0.87 to 0.99. Similarly, *H* ^2^ was also high for BM (0.47–0.89), YLD (0.6–0.99) and HI (0.56–0.99).

### Analysis of variance and trait correlations

The combined ANOVA revealed significant differences among RILs of both populations (ICCRIL03 and ICCRIL04) for all the above-mentioned traits (*P* < 0.05, 0.01 and 0.001; ESM Tables S5 and S6).

In the case of ICCRIL03, significant effect of location was observed for all morphological traits, yield-related traits and block effects were significant for all traits except yield-related traits measured at SEH and DUG location under IR environment. Significant interaction between RILs and location was observed for all traits at 1 % level of significance. The mean square values for 2 years (2005 and 2007) differed significantly from each other for all root traits. Highly significant differences (*P* < 0.001) were found in genotypes (RILs) for all traits except for the trait RV.

Correlation is a pragmatic approach to develop selection criteria for accumulating optimum combination of yield contributing traits in a simple genotype. Among root traits, RTR has significant negative correlation with RL, RLD, and RSA and non-significant correlation with RDp in ICCRIL03 across both seasons (2005 and 2007). δ^13^C, an indirect measure of plant TE has a significant positive correlation with HI and a negative correlation with PHT across locations during 2008, while correlations were non-significant in case of BM, YLD with δ^13^C with all other traits. However, in the case of ICCRIL04, RTR has only negative correlation with SDW. In addition, a non-significant negative correlation was observed between RLD and RTR. Nevertheless, all other traits have significant positive correlation in the case of ICCRIL04 (ESM Table S7).

Furthermore, a significant positive correlation was found between YLD and 100SDW, BM and a negative correlation between YLD and DF, DM, as expected across locations and seasons (ESM Table S7) in both RIL populations.

### Component genetic maps

Screening of 2,717 SSR markers on the parental lines resulted in identification of 321 and 230 polymorphic markers on ICCRIL03 and ICCRIL04, respectively (Lichtenzveig et al. 2005; Sethy et al. 2006; Varshney et al. 2009; Nayak et al. 2010; Gujaria et al. 2011; Gaur et al. 2011; Thudi et al. 2011; ESM Table S2). The genotyping data were generated for the polymorphic markers on respective mapping populations. As a result, 241 marker loci including 214 SSRs, 6 GMMs and 21 DArT loci were placed on to genetic map for ICCRIL03 (Table 2; ESM Figure S1; http://cmap.icrisat.ac.in/cmap/sm/cp/varshney/) and 168 marker loci (151 SSRs, 10 GMMs and 7 DArT loci) in the case of ICCRIL04 (Table 2; ESM Figure S2; http://cmap.icrisat.ac.in/cmap/sm/cp/varshney/). In total, 62 (26.95 %) markers in the case of ICCRIL04 and 80 (24.92 %) markers in the case of ICCRIL03 remained unmapped. Varying levels of marker densities were recorded for different linkage groups (LGs) in both the maps and the average inter-marker distances were 2.71 and 3.27 cM in the case of ICCRIL03 and ICCRIL04, respectively (Table 2). Of 46 markers with segregation distortion, 9 markers were not mapped in case of ICCRIL03, while in the case of ICCRIL04, 20 markers remained unmapped.

**Table 2:** Features of two intra-specific genetic maps and a consensus map

Linkage group (LG)	ICCRIL03 (ICC 4958 × ICC 1882)			ICCRIL04 (ICC 283 × ICC 8261)			Number of common markers between ICCRIL03 and ICCRIL04	Consensus map			Correlation of marker positions with consensus map	
	Markers mapped	Map distance (cM)	Inter-marker distance (cM)	Markers mapped	Map distance (cM)	Inter-marker distance (cM)		Markers mapped	Map distance (cM)	Inter-marker distance (cM)	ICCRIL03	ICCRIL04
CaLG01	31	99.27	3.20	16	60.78	3.80	4	39	107.16	2.75	0.98**	0.98**
CaLG02	18	78.50	4.36	16	66.61	4.16	7	25	75.68	3.03	0.95**	0.98**
CaLG03	41	28.13	0.69	22	69.44	3.16	3	64	70.77	1.11	0.99**	0.99**
CaLG04	45	111.90	2.49	18	43.95	2.44	7	52	111.97	2.15	0.99**	0.98**
CaLG05	22	33.24	1.51	23	51.51	2.24	5	38	58.44	1.54	0.86**	0.95**
CaLG06	36	123.08	3.42	31	65.29	2.11	6	58	119.88	2.07	0.99**	0.98**
CaLG07	27	96.11	3.56	24	104.92	4.37	4	43	155.99	3.63	0.99**	0.98**
CaLG08	21	51.28	2.44	18	70.57	3.92	5	33	71.5	2.17	0.99**	0.99**
Average	30.12	77.68	2.71	21	3.17	3.27	5.12	44	96.42	2.30		
Total	241	621.51		168	533.06		41	352	771.39			

** Significant at 0.01

### QTLs for drought tolerance component traits

To understand the genetic and molecular basis of drought tolerance, developed genetic maps and extensive phenotyping data generated on both RIL populations were analyzed in details for identification of both main-effect QTLs as well as the QTLs showing epistatic interactions.

#### Main-effect QTLs (M-QTLs)

For both RIL populations, M-QTLs were identified using QTL Cartographer and QTLNetwork programs (ESM Figures S1 and S2). In the case of ICCRIL03, QTL Cartographer identified a total of 77 M-QTLs including 36 M-QTLs for yield-related traits; 12 M-QTLs for morphological traits; 11 M-QTLs for root traits; 9 M-QTLs for phenological traits; 7 M-QTLs for drought tolerance indices and 2 M-QTLs for δ^13^C (ESM Table S8). In case if one of two flanking markers is common in more than one QTL, we have considered that region as only one genomic region that contains >1 QTL. By following this criteria, 77 M-QTLs identified were present in 36 genomic regions. On the other hand, QTLNetwork analysis provided 62 M-QTLs in 22 genomic regions. These QTLs include 26 M-QTLs for yield and yield-related traits; 14 M-QTLs for morphological traits; 10 M-QTLs for phenological traits; 5 M-QTLs for root traits; 6 M-QTLs for drought tolerance indices and 1 M-QTL for δ^13^C (ESM Table S9). Of the 77 M-QTL detected by QTL Cartographer, nearly 40 % of the QTLs (30 M-QTLs) were located on CaLG04 followed by CaLG01 (12 M-QTLs). The similar observations were made in QTLNetwork analysis in which 28 of 62 M-QTLs were present on CaLG04 followed by CaLG01 (14 M-QTLs).

In the case of ICCRIL04, 51 M-QTLs in 25 genomic regions were identified by QTL Cartographer, which include 15 M-QTLs for yield-related traits, 14 M-QTLs for phenological traits, 11 M-QTLs for morphological traits, 7 M-QTLs for root-related traits and 4 M-QTLs for drought indices (ESM Table S8). QTLNetwork detected 13 M-QTLs in ten genomic region and includes 5 M-QTLs for phenological traits, 4 M-QTLs for morphological traits, 3 M-QTLs for yield-related traits and 1 M-QTL for DTI (ESM Table S9; ESM Figure S2). Majority of QTLs identified by any of these programs were located on CaLG01 followed by CaLG08, CaLG06.

#### Epistatic QTLs (E-QTLs)

For understanding the complexity of drought tolerance traits, QTLNetwork and genotype matrix mapping program (GMM program) were used to detect E-QTLs in both RILs. For instance in the case of ICCRIL03, by considering two loci interactions, a total of 26 E-QTLs were identified that include 15 E-QTLs detected by QTLNetwork (ESM Table S10) and 11 detected by GMM program (ESM Table S11). These QTLs contribute up to 26.18 % phenotypic variation for 10 of 20 traits phenotyped and DTI. GMM program also provided 693 E-QTLs by considering three loci interactions for all the 20 traits and both drought indices with 7.09–91.56 % phenotypic variation (ESM Table S11).

Similarly in the case of ICCRIL04, a total of 13 E-QTLs were detected by QTLNetwork (ESM Table S10) and no QTL was detected by GMM program for two loci interaction (ESM Table S12). These QTLs contribute from 3.57 to 13.25 % phenotypic variation for 7 of 20 traits phenotyped and DTI. GMM program also provided 295 E-QTLs by considering three loci interactions for 16 traits and drought indices with 0.49–92.19 % phenotypic variation (ESM Table S12).

### Trait dissection

Comprehensive QTL analysis of both M-QTLs and E-QTLs provided an opportunity to analyze drought tolerance component traits in depth. As QTL analysis was undertaken on phenotypic data for 20 traits and 2 drought indices, collected in 1–7 years (seasons) at 1–5 locations, phenotypic data collected in a given year at given location were considered as one environment. By considering this criterion, QTL analysis was undertaken for 20 traits across 20 environments. Phenotypic variation explained (PVE) by M-QTLs ranged from 2.34 to 58.20 % in case of ICCRIL03 and 2.95 to 31.32 % in case of ICCRIL04 (ESM Table S8), while E-QTLs explained 0.75 to 91.56 % PVE in ICCRIL03 and 0.49 to 92.19 % PVE in the case of ICCRIL04 (ESM Tables S11 and S12). For trait dissection in comprehensive manner, only robust M-QTLs and E-QTLs that contribute >10 % PVE were considered into account. If the QTL for a given trait appeared in more than one location, it was considered as ‘stable’ QTL and if this appears in more than 1 year/season, the QTL was considered as ‘consistent’ QTL.

Furthermore, a quick comparison of M-QTLs identified by QTL Cartographer and QTLNetwork showed that M-QTLs detected by QTL Cartographer include all or key QTLs detected by QTLNetwork; therefore, M-QTLs identified by QTL Cartographer only were considered for trait dissection analysis. Similarly, GMM program analysis provided more comprehensive E-QTLs (both two loci as well as three loci interactions) as compared to QTLNetwork, and thus GMM program analysis-based E-QTLs with 3 loci interactions were included for trait dissection analysis.

In brief, M-QTLs detected by QTL Cartographer with >10 % PVE and E-QTLs (3 loci interactions) detected by GMM program with >10 % PVE were used for comprehensive genetic analysis of drought tolerance component traits.

#### Root traits

Of the six root traits analyzed, robust M-QTLs were identified for three traits one each for RLD, RSA and RTR in ICCRIL03 (Table 3) and therefore no stable and consistent QTL was detected. In terms of robust E-QTLs, robust 3 loci epistatic interactions were observed for all six traits (Table 4). For instance, for RLD, one E-QTL [TA127 (BB) TA180 (BB) ICCM0065 (BB)] contributing 31.41 % PVE was observed in 2005. In 2007, although eight robust E-QTLs contributing from 23.5 to 33.23 % PVE were identified, one locus, namely TA180, was common in robust E-QTLs of 2005 as well as in all eight robust E-QTLs of 2007 (ESM Table S11).

**Table 3:** Main-effect QTLs (M-QTLs) for drought tolerance related traits identified in two RIL populations

Trait	ICCRIL03 (ICC 4958 × ICC 1882)			Phenotypic variation explained (PVE, %)		ICCRIL04 (ICC 283 × ICC 2861)			Phenotypic variation explained (PVE, %)
	No. of QTLs	Stable QTLs	Consistent QTLs			No. of QTLs	Stable QTLs	Consistent QTLs	
Root									
RLD	1	–	–	10.90		–	–	–	–
RSA	1	–	–	10.26		–	–	–	–
RTR	1	–	–	16.67		–	–	–	–
Morphological									
SDW	1	–	1	13.89–17.59		–	–	–	–
PHT	4	1	2	10.00–30.20		2	–	1	11.27–31.32
PWD	–	–	–	–		1	–	–	15.84
Phenological									
DF	2	1	1	10.51–26.87		4	1	2	10.66–18.97
DM	3	1	1	12.13–19.71		4	1	–	10.47–16.79
Yield related									
100SDW	2	1	1		10.31–58.20	1	–	1	17.14–26.67
BM	2	–	–		10.95–21.32	–	–	–	–
HI	3	–	–		10.67–14.36	2	–	–	12.06–14.04
POD	1	–	1		10.19–23.18	1	–	1	12.13–14.37
SPD	1	–	–		42.07	–	–	–	–
YLD	2	–	–		13.98–15.71	3	–	–	10.06–18.55
Drought indices									
DTI	1	–	–		11.23	2	–	–	11.27–12.12
Total	25	4	7			20	2	5

**Table 4:** Summary on three loci epistatic interactions in two RIL populations based on genotype matrix mapping program (GMM program) analysis

Traits	ICCRIL03		ICCRIL04	
	Three loci interactions		Three loci interactions	
	No. of QTLs	PVE^a^ (%)	No. of QTLs	PVE^a^ (%)
Root				
RLD	9	23.49–33.23	9	13.25–44.20
RDW	2	17.77–20.72	11	18.03–44.61
RDp	12	10.71–24.39	–	–
RSA	11	14.93–42.97	–	–
RTR	4	23.25–34.99	4	17.82–22.60
RV	16	16.61–19.53	3	29.37–36.61
Morphological				
SDW	11	12.80–21.76	3	21.12–76.26
PHT	39	14.36–76.54	31	14.63–69.50
PWD	3	23.35–37.28	3	13.46–16.06
PBS	13	12.70–28.45	1	32.53
SBS	5	13.26–27.93	3	36.92–44.41
Phenological				
DF	70	10.80–81.21	7	16.63–61.23
DM	150	13.44–91.55	15	15.12–56.34
Yield related				
100SDW	7	11.86–22.46	55	11.23–80.55
BM	86	10.63–35.47	44	11.54–63.51
HI	63	11.02–54.28	41	16.19–81.58
POD	13	12.12–22.80	4	27.77–59.30
SPD	43	10.98–35.91	–	–
YLD	82	10.04–54.36	34	20.50–92.19
Transpiration related				
δ^13^C	2	16.89–43.10	–	–
Drought indices				
DSI	19	11.61–28.63	3	70.09–80.95
DTI	26	15.64–41.28	16	15.04–91.83
Total	686		287	

^a^ *PVE* phenotypic variation explained

In the case of ICCRIL04, although no robust M-QTL was identified for any trait, 3–11 robust E-QTLs with up to 44.61 % PVE were identified for RLD, RDW, RTR and RV traits (Table 4). In majority of the cases, identified robust E-QTLs were consistent, as at least one locus present in a robust E-QTL identified in 1 year was also present in the robust E-QTL identified in the other year.

#### Morphological traits

Of the five traits analyzed for morphological characters, five robust M-QTLs (4 for PHT and 1 for SDW) with up to 30.20 % PVE were identified for two traits (SDW and PHT) in ICCRIL03 (Table 3). Of these five robust M-QTLs, a QTL named ‘*QR3sdw01*’ flanked by ‘TAA170–NCPGR21’ on CaLG04 appeared consistently for two seasons (2005 and 2007) for SDW. Further, two QTLs for PHT (‘*QR3pht01*’ flanked by ‘CaM1760–CaM0399’ on CaLG06 and ‘*QR3pht03*’ flanked by ‘NCPGR127–NCPGR21’ on CaLG04) were consistent in 2005, 2007, 2008 and 2009. In addition, the QTL ‘*QR3pht03*’ was stable across 1–5 locations (PAT, SEH, NDL, DUG and HIR) (ESM Table S8). In terms of epistatic interactions, 71 robust E-QTLs with up to 76.54 % PVE were detected for all 5 traits analyzed (Table 4; ESM Table S11). Majority of these QTLs have at least one common locus interacting with other two loci (ESM Table S11).

In the case of ICCRIL04, three robust M-QTLs (2 for PHT and 1 for PWD) with up to 31.32 % PVE were detected for PHT and PWD. Of these three robust M-QTLs, one QTL ‘*QR4pht02*’ flanked by ‘CaM0772–TS45’ on CaLG08 consistently appeared in two seasons (2005 and 2006). Furthermore, 41 robust E-QTLs with up to 76.26 % PVE were detected for all five traits (Table 4). Interestingly, one locus ‘TA127’ was observed in 11 of 31 robust E-QTLs identified for PHT (ESM Table S12).

#### Phenological traits

A total of five robust M-QTLs (3 M-QTL for DM and 2 M-QTL for DF) with up to 26.87 % PVE were detected for DF and DM in the ICCRIL03. Of these five M-QTLs, in case of DF, ‘*QR3df01’* QTL flanked by ‘NCPGR164–CaM1918’ on CaLG08 was consistent in three seasons (2005, 2008 and 2009) and stable at four locations (PAT, HIR, NDL and DUG). While, in the case of DM, the *QR3dm01* flanked by ‘NCPGR164–CaM1918’ on CaLG08 was consistent for two seasons (2008 and 2009) and stable at three locations (PAT, HIR and DUG) (Table 3; ESM Table S8). In addition, although a large number of robust E-QTLs (220) were detected for all traits studied, one locus, namely ‘NCPGR203’, had the highest interaction in 21 E-QTLs for DM (ESM Table S11).

In the case of ICCRIL04, eight robust M-QTLs (4 each for DF and DM) were identified with up to 18.97 % PVE. In case of DF, one QTL ‘*QR4df01’* flanked by ‘CaM1753–cpPb-677529’ on CaLG03 was consistent across two seasons (2005 and 2006) and another QTL ‘*QR4df06*’ flanked by ‘TA103II–TA122’ was consistent across two seasons (2010 and 2011) and stable across two locations (HIR and NDL). In the case of DM, one QTL ‘*QR4dm05’* flanked by ‘TA103II–TA122’ on CaLG01 was stable at two locations (PAT and HIR) (Table 3; ESM Table S8). In addition, 22 E-QTLs with up to 61.23 % PVE were detected for both the traits (Table 4).

#### Yield and yield-related traits

The QTL analysis of six yield-related traits detected a total of 11 robust M-QTLs (3 for HI, 2 each for 100SDW, BM and YLD, 1 each for POD and SPD) which explained up to 58.20 % PVE in ICCRIL03. For 100SDW, interestingly one QTL ‘*QR3100sdw03*’ flanked by ‘NCPGR127–NCPGR21’ on CaLG04 was consistent across three seasons (2006, 2008 and 2009) and stable across all the five locations (PAT, HIR, SEH, NDL and DUG). Further, for POD QTL ‘*QR3pod01’* flanked by ‘NCPGR127–NCPGR21’ on CaLG04 was consistent across four seasons (2005, 2006, 2007 and 2009) (Table 3; ESM Table 8). Further in terms of E-QTLs, 294 robust E-QTLs explained up to 54.37 % PVE (for 6 traits) in the case of ICCRIL03. Among 294 robust E-QTLs detected, one locus, namely ‘TAA170’, showed interaction in 21 of 82 robust E-QTLs for yield (ESM Table 11).

In the case of ICCRIL04, seven robust M-QTLs were detected (3 for YLD, 2 for HI and 1 for 100SDW and POD); of these QTL for 100SDW ‘*QR4100sdw02’* flanked by ‘CaM2093–ICCM0249’ on CaLG04 was consistent across three seasons (2005, 2006 and 2007) and one QTL for POD ‘*QR4pod02’* flanked by ‘CaM0772–TS45’ on CaLG08 was consistent across two seasons (2006 and 2007). A total of 178 robust E-QTLs were identified (Table 4).

#### Transpiration efficiency

No robust M-QTL for δ^13^C was detected in the case of ICCRIL03, while in the case of ICCRIL04, δ^13^C was not measured. Only two robust E-QTLs with 16.89 to 43.10 % PVE were identified in ICCRIL03 (Table 4). This indicates that minor QTLs, showing interaction, play a significant role for TE.

#### Drought tolerance and susceptible indices

In case of ICCRIL03, one robust M-QTL with 11.23 % PVE and 45 robust E-QTLs with up to 41.28 % PVE were identified for DTI (Tables 3, 4). Among robust 45 E-QTLs, one locus, namely ‘ICCM0257’, was interacting among 7 QTLs identified in 2009 (ESM Table 11). In the case of ICCRIL04, two robust M-QTLs with 11.27–12.12 % PVE and 19 E-QTLs with up to 91.83 % PVE were identified (Tables 3, 4). No consistent and stable QTLs were observed for DTI and DSI drought tolerance indices in both RIL populations.

### Consensus genetic and QTL map

**Fig. 1:**
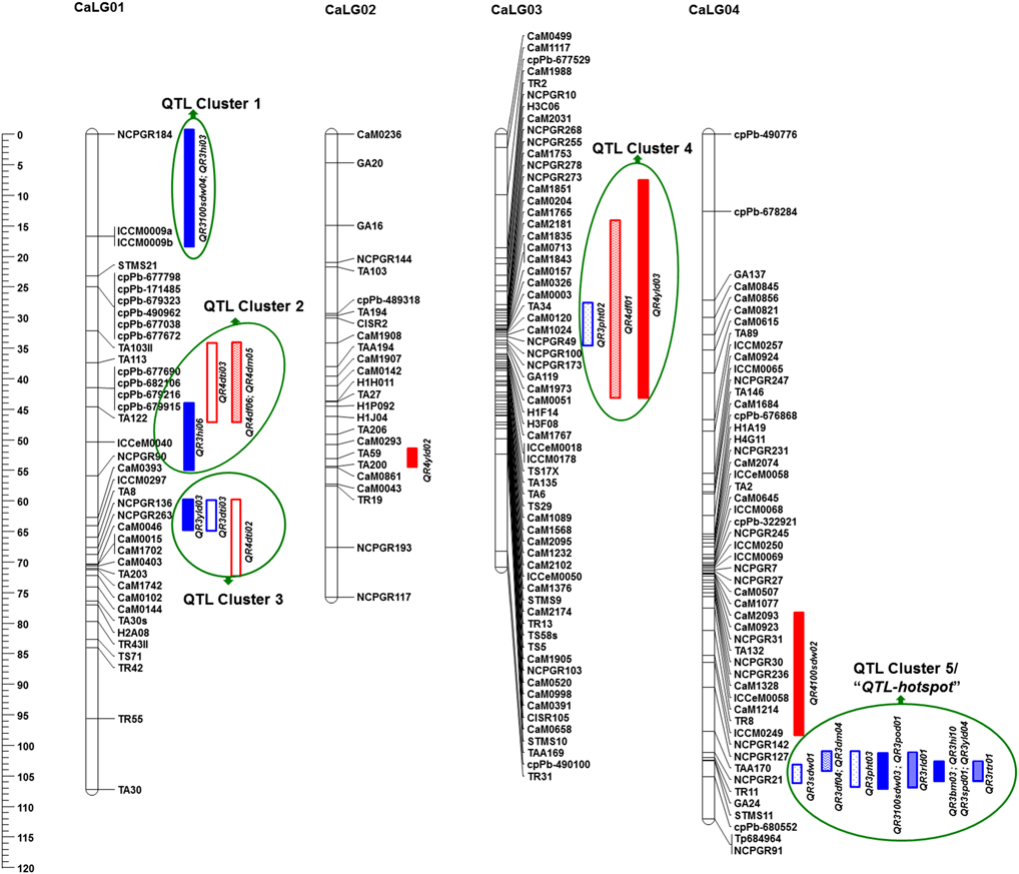

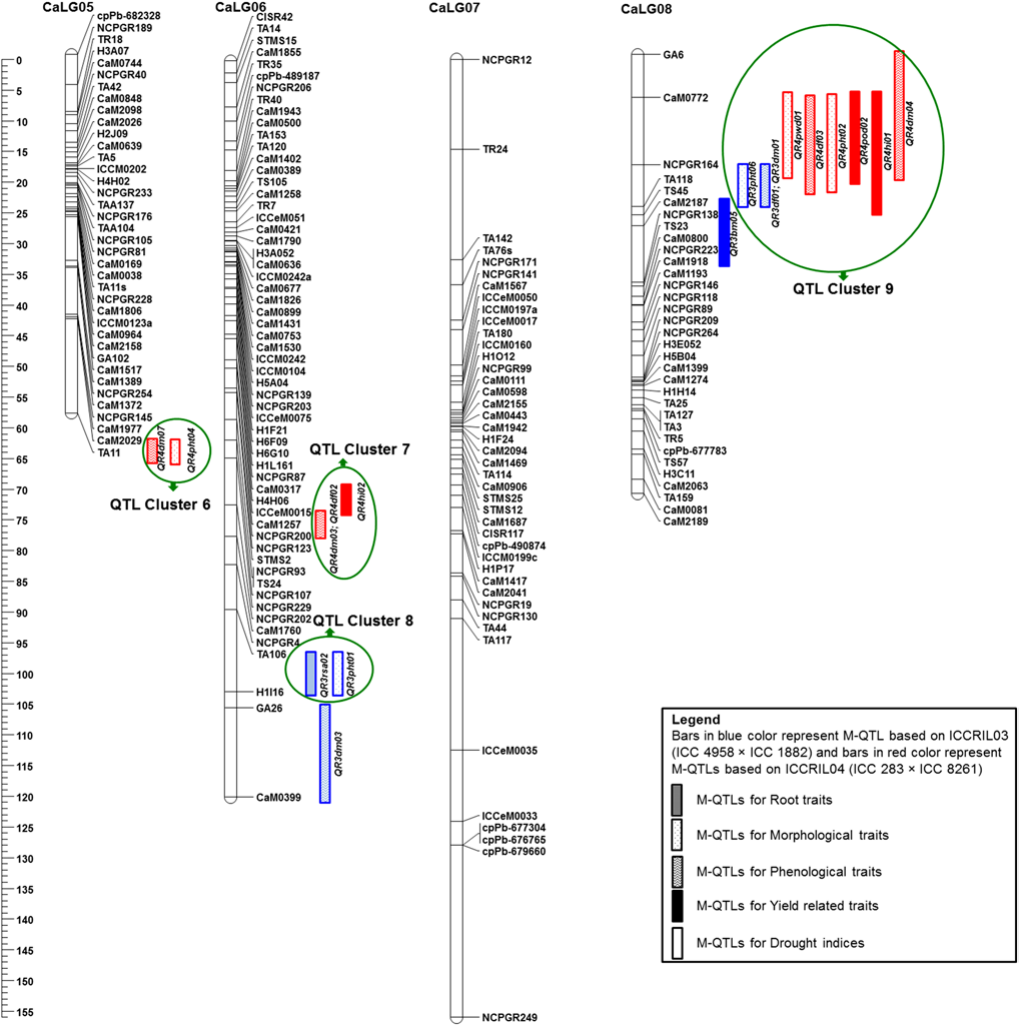
Consensus genetic and QTL map comprising 352 marker loci based on two intra-specific mapping populations. Markers are shown on the *right side* of the LG, while map distances are shown on the *left side*. The QTLs identified from the ICCRIL03 and ICCRIL04 populations are differentiated by *different colors*

While comparing two intra-specific genetic maps, 43 marker loci were found common between two maps. These markers were considered as anchor markers and used for merging the genetic maps for construction of consensus genetic map. The consensus map comprised 352 loci and covered a total map distance of 771.39 cM. The length of LGs ranged from 58.44 cM (CaLG05) to 155.99 cM (CaLG07) (Table 2; Fig. 1). The density of markers on the map ranged from 1.11 cM/marker on CaLG3 to 3.63 cM/marker on CaLG07, with an average density of 2.30 cM/marker. However, 6.36 % (26) markers could not be integrated on to the consensus genetic map. Among different types of marker (SSR/STMS, EST-SSR, conserved intron spanning regions, CISR; cleaved amplified polymorphic sequence (CAPS) and DArT) loci, the consensus genetic map predominantly consists of SSR marker loci (322). Majority of the DArT loci (40 %) were confined to CaLG01, while the remaining DArT loci were mapped on CaLG07 and CaLG04. Of five CAPS markers used for mapping, only one marker (Tp684964) was mapped on CaLG04.

**Fig. 2:**
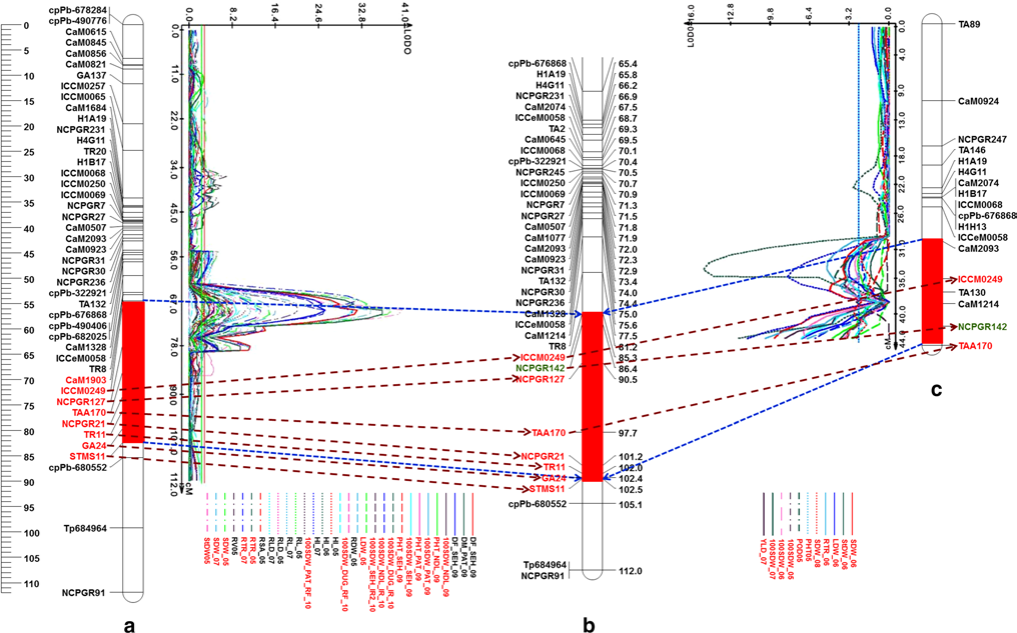
Comparison of “*QTL-hotspot*” genomic region harboring QTLs for various drought tolerance-related traits identified on CaLG04 of two intra-specific mapping populations with genomic region on consensus map. **a** QTLs identified based on ICCRIL03 (ICC 4958 × ICC 1882) mapping population. **b** CaLG04 of consensus genetic map. **c** QTLs identified based on ICCRIL04 (ICC 283 × ICC 8261) mapping population. QTLs common to traits in both mapping populations are highlighted in *red*

Detailed comparison using CMap among consensus map and population specific/component genetic maps revealed a very high congruency in terms of marker orders corresponding LGs and markers grouping on LGs (correlation coefficients varying from 0.86 to 0.99; Table 2). An example of correlation in one linkage group (CaLG04) has been shown in Fig. 2. The CaLG04 has seven markers common between two component genetic maps and six of these seven markers were placed on the consensus map. The figure shows a good conservation of marker order amongst consensus and the two genetic maps. Comparison of each LG across three maps can be visualized at http://cmap.icrisat.ac.in/cmap/sm/cp/varshney/.

Efforts were also made to place the detected robust M-QTLs as mentioned earlier on the consensus map. All 25 and 20 robust M-QTLs detected in ICCRIL03 and ICCRIL04, respectively, were placed on the consensus map. These 45 robust M-QTLs for 14 traits and DTI contribute up to 58.20 % PVE.

Genomic regions containing QTLs for several traits are much valued by breeders. In this context, we analyzed the detected QTLs and considered QTL cluster/co-localized QTLs if they represent for more than three traits. In case of ICCRIL03, two QTL clusters (each one on CaLG04 and CaLG08) were identified. A QTL cluster on CaLG04 co-localized 12 QTLs influencing 12 traits (RLD, RTR, SDW, DF, DM, SPD, PHT, POD, HI, YLD, BM and 100SDW) with up to 58.20 % PVE (Fig. 1). Similarly, a cluster on CaLG08 clustered four QTLs influencing four traits (DF, DM, BM and PHT) with up to 26.87 % PVE.

In the case of ICCRIL04, QTLs were co-localized on CaLG08. A total of six QTLs for six traits (DF, DM, PHT, POD, HI and PWD) with up to 31.32 % PVE were co-localized between GA6 and NCPGR138 markers on CaLG08 (ESM Fig. 2). Furthermore, mapping of QTLs identified in two RIL populations on the consensus genetic map provided nine QTL clusters (Fig. 1). Among nine QTL clusters, QTL Cluster 1, QTL Cluster 2 and QTL Cluster 3 were located on CaLG01; QTL Cluster 4 on CaLG03, QTL Cluster 5 on CaLG04; QTL Cluster 6 on CaLG05; QTL Cluster 7 and QTL Cluster 8 were on CaLG06 and QTL Cluster 9 was on CaLG08.

### “*QTL-hotspot*” region for drought tolerance

While analyzing robust M-QTLs in detail, an interesting genomic region (29 cM) containing seven markers (ICCM0249, NCPGR127, TAA170, NCPGR21, TR11, GA24 and STMS11) was identified on CaLG04 of genetic map for ICCRIL03. This region contained 12 out of 25 (48 %) robust M-QTLs for 12 traits (100SDW, RLD, DF, DM, BM, PHT, POD, HI, RTR, SDW, SPD and YLD). Furthermore, one consistent QTL each for SDW, PHT, POD and 100SDW and one stable QTL each for PHT and 100SDW were located in this genomic region (Table 3).

Similarly, one genomic region in the case of ICCRIL04, spanning 15 cM with six markers (CaM2093, ICCM0249, TA130, CaM1214, NCPGR142 and TAA170) was identified on CaLG04 of the genetic map of ICCRIL04. However, only one consistent QTL was observed in this genomic region and none of the identified QTLs were stable.

While comparing genomic regions on CaLG04 of component genetic maps of two populations, two markers (ICCM0249 and TAA170) were found common in the regions. Therefore, the regions identified in two-component genetic maps are the same region in the chickpea genome. This region is the QTL Cluster 5. As this region contained a total of 13 robust QTLs for 12 traits with 58.20 % PVE and identified in genetic maps of both RILs, we have designated this region as “*QTL-hotspot*” region in chickpea genome. As this “*QTL-hotspot*” contained four consistent and two stable QTLs for 12 traits and DTI with up to 58.20 % PVE in ICCRIL03 and one consistent QTL with up to 26.68 % PVE in ICCRIL04, this region can be considered as a promising drought tolerance candidate genomic region for molecular breeding.

## Discussion

Towards understanding complexity of drought tolerance in chickpea, a few expression and functional genomics (Varshney et al. 2009; Deokar et al. 2011) and physiological (Zaman-Allah et al. 2011) studies were conducted in recent past; however, the genetics and molecular mechanisms for drought tolerance is still not well understood. This study reports genetics-based dissection of drought tolerance after generating and analyzing extensive phenotyping and genotyping data on two segregating populations.

### Extensive and precise phenotyping for drought tolerance

To better understand drought tolerance mechanism in chickpea, 20 drought tolerance component traits were phenotyped under 1–7 seasons at 1–5 locations in India. Detailed analysis of phenotyping data on six root traits indicated that the phenotypic variation among RILs in the ICCRIL03 population was almost double for all root traits studied compared to earlier studies (Serraj et al. 2004; Kashiwagi et al. 2008), although earlier studies deployed germplasm and RILs were studied in the present study. In the case of ICCRIL04, although variation among RILs was high, the variation between parental genotypes was comparatively low than that of the ICCRIL03. The broad sense heritability (*H* ^2^) for the six root traits ranged from 0.07 to 0.61 in the ICCRIL03 and 0.15–0.48 in ICCRIL04. As reported earlier (Kashiwagi et al. 2005), the *H* ^2^ was high in case of RLD. Since RLD is associated with greater yield under terminal drought conditions, selection for such a trait with high *H* ^2^ in breeding may help enhancing the genetic gains and yield improvement in chickpea. The phenological traits such as DF and DM possessed high *H* ^2^ across locations and in different environments/seasons, indicating that selection for these traits will also be effective in breeding. Among yield-related traits, the high *H* ^2^ was observed in case of 100SDW across locations, seasons/environments indicating that 100SDW is the least affected trait by the environment and selection for this trait may positively improve yield under terminal drought conditions.

### Genetic and consensus maps

Most of the dense genetic maps developed to date in chickpea are based on inter-specific crosses (Nayak et al. 2010; Thudi et al. 2011; Gaur et al. 2012; Hiremath et al. 2012). Although intra-specific genetic maps (Radhika et al. 2007; Gaur et al. 2011) as well as consensus maps based on intra-specific crosses were developed in chickpea (Radhika et al. 2007; Millàn et al. 2010; Gaur et al. 2011), marker density was very low and maximum number of marker loci (including random amplified polymorphic DNA, RAPD and sequence tagged microsatellites, STMS) mapped on to a single intra-specific genetic map are 138 (Gaur et al. 2011) and the consensus map has 229 markers (Millàn et al. 2010). The present study reports a significant improvement of marker density in the intra-specific component (2-fold) and consensus (1.5-fold) genetic maps. Consensus map reported here comprised 352 marker loci across all 8 LGs, spanning a total distance of 771.39 cM and is developed based on two intra-specific RIL populations. Unlike other published maps for intra-specific mapping populations which contained anonymous markers (like RAPD, AFLP), the consensus map developed in the present study comprised mainly SSR markers. Marker order and marker distribution on individual genetic maps as well as consensus map were highly conserved (*P* ≤ 0.98; Table 2). Furthermore, comparison of the consensus map of the present study with the inter-specific map developed by Thudi et al. (2011) also revealed a high conservation of marker order and 38 markers were common between these two genetic maps. Sparse distribution of marker loci towards telomeres in the cases of CaLG01, CaLG02 and CaLG07 may be due to lower recombination rates and such kind of low marker densities in telomeric regions was also observed in earlier studies (Nayak et al. 2010; Thudi et al. 2011). Similarly, higher genomic SSR marker density towards the centromeres indicates the unequal recombination rates among the chickpea chromosomes.

High congruency in terms of marker order observed in case of component genetic maps and consensus map in the present study will be quite useful for ordering future genetic maps. Higher marker density of the consensus map, compared to other published maps (Millàn et al. 2010), will allow selection of specific markers for molecular breeding applications such as fine mapping, the development of novel genetic stocks (e.g., near isogenic lines and inbred backcross lines). This consensus map will also provide opportunities of anchoring with the physical map and facilitate mapping of known genes from legumes based on synteny.

### Simplification of complex traits

In the present study, a large number of QTLs for several drought component traits have been identified by CIM analysis. Although QTL Cartographer, QTLNetwork and GMM program were used for detailed analysis, M-QTLs identified by QTL Cartographer and E-QTLs (3 loci interactions) identified by GMM program have been considered for further analysis. In order to gain deeper insights into drought tolerance, five groups of drought tolerance-related traits, namely root traits, morphological traits, phenological traits, yield and yield-related traits and TE, were attempted for genetic and molecular dissection (Ravi et al. 2011).

For six root traits analyzed in two RIL populations, a total of 18 M-QTLs were identified on all LGs except CaLG02. While considering only robust QTLs, 3 M-QTLs one each for RLD (CaLG04), RSA (CaLG06) and RTR (CaLG04) were found specific to only ICCRIL03. As ICCRIL03 and ICCRIL04 have ICC 4958 and ICC 8261 drought tolerant parents, ICC 4958 seems to have major-effect QTLs for identified root traits. ICC 8261 either has only small-effect QTLs or robust QTLs present in the ICC 8261 could not be identified in this study. On the other hand, all identified 81 E-QTLs were robust and present in both RIL populations that indicate that epistatic interaction plays a significant role in expression of root traits.

In case of morphological traits, a total of 23 M-QTLs were found on all LGs except CaLG02. These QTLs included eight robust M-QTLs on five LGs for three traits, namely SDW (CaLG04), PHT (CaLG03, CaLG04, CaLG05, CaLG06 and CaLG08) and PWD (CaLG08). QTLs for SDW and PWD were specific to populations. However, both populations share at least one QTL for PHT on CaLG08 that contribute 14.73 % PVE (ICCRIL03) to 31.32 % PVE (ICCRIL04). In addition, 112 E-QTLs were robust QTLs with up to 76.54 % PVE on all LGs indicating prominent role of epistatic interaction in expression of morphological traits.

For phenological traits, a total of 23 M-QTLs on all LGs except CaLG02 including 13 robust M-QTLs were detected in two RIL populations for both traits (DF and DM). Occurrence of 13 robust M-QTLs on 6 LGs indicates quantitative nature of the traits. Furthermore, identification of 242 E-QTLs present all over the genome highlights the involvement of epistatic interaction for phenological traits.

Yield is considered to be important trait for chickpea farmers in semi-arid regions where terminal drought is prevailing. A total of 51 M-QTLs and 480 E-QTLs were identified for 6 yield and yield-related traits in two RIL populations. However, only 18 M-QTLs present on all LGs except CaLG05 and CaLG07, and 472 E-QTLs present on all LGs were robust. As all 18 M-QTLs are specific to one of two populations and contribute a range of phenotypic variation, yield and yield-related traits show the complex nature of genetics.

For TE-related traits, a total of 2 M-QTLs (both on CaLG04) and 2 E-QTLs were found in the ICCRIL03 of which only E-QTLs were robust. Low number of QTLs identified in the study is a function of use of smaller set of phenotyping data obtained in only one population and in 1 year as compared to datasets for other traits.

### Candidate genomic regions for molecular breeding

In any breeding program, the traits to be considered as potential selection targets for improving yield under water-limited conditions must be genetically correlated with yield, and should have a greater *H* ^2^ than yield itself (Blum 2011). As mentioned earlier, root traits are drought avoidance traits, phenological traits (DF and DM) are drought escape traits and WUE or TE is drought tolerance traits. Improving any one or combination of these traits will improve yield under drought conditions (Gaur et al. 2008). Of course, yield and yield-related traits like HI under drought conditions are the ultimate targets in a breeding program (Krishnamurthy et al. 2013b).

The present study reports nine QTL clusters that have robust QTLs for all of the above-mentioned traits except TE. For instance, QTL Cluster 5 contains QTLs for root traits (RLD 10.90 % PVE, RTR 16.67 % PVE), phenological traits (DF 24.49 % PVE, DM 19.71 % PVE) as well as yield and yield-related traits (100SDW 58.20 % PVE, POD 23.18 %, BM 21.32 %, SPD 42.07 %, HI 11.69 %). In fact, this region has been referred as “*QTL-hotspot*” as this region contained several stable and consistent QTLs with higher PVE. While considering all the QTLs, this region contains 17 (22.07 % of total) QTLs for 15 traits including TE and DTI were identified in ICCRIL03 that contributes from 4.49 to 58.2 % PVE. Of these QTLs, four were consistent and two were stable QTLs. Furthermore, seven QTLs for five traits identified in the ICCRIL04 also fell in the same region. In brief, this region has 22 QTLs for 15 traits for all the five groups of traits analyzed across two RILs. Therefore, this region seems to be of utmost importance for introgression in elite varieties for enhancing yield under drought conditions.

In addition to above, QTL Cluster 9 present on CaLG08 also seems to be an interesting genomic region for targeting for molecular breeding as it contains QTLs for DF (26.87 %), DM (18.83 %), HI (14.04 %), PHT (31.32 %), PWD (15.84 %) and POD (14.38 %). Hence, introgression of this cluster will not only improve the component traits and but also yield in chickpea under drought as it improves HI, a key component trait for estimating yield under drought (Passioura 1977). Introgression of QTL Cluster 1, QTL Cluster 5, QTL Cluster 7 and QTL Cluster 8 will improve HI in total. Larger RSA will enhance soil contact and enable absorption of more available water, thus avoiding drought. Introgression of QTL Cluster 7 on CaLG06 simultaneously improves both drought escape traits like DM and drought avoidance traits like RSA.

Furthermore, large number of epistatic QTLs for different traits identified in present study indicates that the QTLs with minor effects/no effect interact with the other loci and influence the expression of the traits. For instance, although no robust QTL was identified for RLD, an important drought avoidance trait, in the genetic background of ICCRIL04, nine epistatic interactions were identified with up to 44.21 % PVE. Nevertheless, epistatic interactions with high phenotypic variation were identified for the traits like RDW, RV and RDp, although no robust QTLs were identified across any genetic background. Further, for δ^13^C an indirect measure of TE, which in turn is an important trait for estimating yield under drought conditions (Krishnamurthy et al. 2013a), no robust QTL was detected; however, epistatic interactions with up to 43.10 % PVE were identified in the case of ICCRIL03. Therefore, for harnessing such epistatic interactions, genomic selection (GS) will be the best alternative in achieving larger genetic gains in shorter periods (Varshney et al. 2012).

## Conclusion

The present study reports on the development of two intra-specific genetic maps of chickpea that were integrated into a single consensus map containing 352 markers, with an average marker density of 2.3 cM/marker, increasing dramatically the density over previously published genetic maps. The consensus map with QTLs integrated will be a valuable resource that will prompt the chickpea research community for next generation genomic and genetic studies. This study also provides nine QTL clusters containing QTLs for all target traits—drought avoidance, drought escape and drought tolerance. Among these QTL clusters, the QTL Cluster 5 on CaLG04, referred as “*QTL-hotspot*” harboring stable and consistent QTLs for several drought tolerance traits, is the most significant region in molecular breeding for improving yield under terminal drought conditions (Varshney et al. 2013). In addition, there are several other QTL clusters that either individually or in combination can be target for introgressing or pyramiding superior alleles for drought tolerance in elite varieties. Analysis of QTL map with genome sequence has suggested the length of the “*QTL-hotspot*” as 7.74 Mb region in the genome. This region contains few hundred genes. Availability of high-throughput sequencing technologies offers the possibility to fine map and eventually clone the QTL region, e.g., “*QTL-hotspot*”, and identify the candidate genes or/and transcription factors to understand the molecular mechanisms for drought tolerance.

## Electronic supplementary material

Below is the link to the electronic supplementary material.

ESM Table S1: Summary of traits evaluated at different locations and seasons on both RIL populations (XLSX 11 kb)

ESM Table S2: Number of polymorphic markers genotyped and mapped on two intra-specific genetic maps (XLSX 9 kb)

ESM Table S3: Mean performance of the parents, variation in RILs, broad sense heritability across seasons and locations in ICCRIL03 (ICC 4958 × ICC 1882) (XLSX 21 kb)

ESM Table S4: Mean performance of the parents, variation in RILs, broad sense heritability across seasons and locations in ICCRIL04 (ICC 283 × ICC 8261) (XLSX 21 kb)

ESM Table S5: Analysis of variance for root, morphological, phenological, yield and yield related, transpiration efficiency related traits on ICCRIL03 (ICC 4958 × ICC 1882) (XLSX 18 kb)

ESM Table S6: Analysis of variance for root, morphological, phenological, yield and yield related traits on ICCRIL04 (ICC 283 × ICC 8261) (XLSX 17 kb)

ESM Table S7: Summary of combined correlations for different drought component traits. a. Combined correlations among root traits based on phenotyping of ICCRIL03 during post-rainy 2005 and 2007 and ICCRIL04 during post-rainy 2006 and 2010. b. Combined correlations among morphological, phenological, transpiration efficiency related, yield and yield related traits phenotyped on ICCRIL03 during post-rainy 2008 at Patancheru, Nandyal, Sehore and Durgapura. c. Combined correlations among morphological, phenological, transpiration efficiency related, yield and yield related traits phenotyped on ICCRIL03 during post-rainy 2009 at Patancheru, Nandyal, Sehore, Hiriyur and Durgapura. d. Combined correlations among morphological phenological transpiration efficiency related, yield and yield related traits phenotyped on ICCRIL04 during post-rainy 2010 at Patancheru, Nandyal, Sehore and Durgapura (XLSX 12 kb)

ESM Table S8: Summary of main-effect QTLs (M-QTLs) for various drought tolerance related traits identified using QTL Cartographer on ICCRIL03 and ICCRIL04 (XLSX 28 kb)

ESM Table S9: Summary of main-effect QTLs (M-QTLs) for various drought tolerance related traits identified using QTLNetwork on ICCRIL03 (ICC 4958 × ICC 1882) and ICCRIL04 (ICC 283 × ICC 8261) (XLSX 24 kb)

ESM Table S10: Summary of epistatic QTLs for various drought tolerance related traits identified using QTLNetwork on ICCRIL03 (ICC 4958 × ICC 1882) and ICCRIL04 (ICC 283 × ICC 8261) (XLSX 14 kb)

ESM Table S11: Two and three loci epistatic interactions identified by Genotype Matrix Mapping program (GMM program) on ICCRIL03 (ICC 4958 × ICC 1882) (XLSX 126 kb)

ESM Table S12: Two and three loci epistatic interactions identified by Genotype Matrix Mapping program (GMM program) in ICCRIL04 (ICC 283 × ICC 8261) (XLSX 40 kb)

ESM Figure S1: The intra-specific genetic map and QTL maps of chickpea constructed based on recombinant inbred line (RIL) mapping population ICC 4958 × ICC 1882 with 241 loci spanning 621.51 cM. The genetic distance in cM is represented on left hand side and the markers names are on the right hand side of the linkage group (TIFF 13128 kb)

ESM Figure S2: The intra-specific genetic map and QTL maps of chickpea constructed based on recombinant inbred line (RIL) mapping population ICC 283 × ICC 8261 with 168 loci spanning 533.06 cM. The genetic distance in cM is represented on left hand side and the markers names are on the right hand side of the linkage group (TIFF 13613 kb)

## Supplementary Figures on CMap site

The CMap site http://cmap.icrisat.ac.in/cmap/sm/cp/varshney/ presents following, in addition to supplementary material of this article (ESM Table S1 to ESM Table S12 and ESM Figure S1 to ESM Figure S2): i) Consensus genetic map with 352 loci, ii) Correspondence of different linkage groups (LG1 to LG8) of Thudi et al. (2011) and two intra-specific maps (ICC 4958 × ICC 1882 and ICC 283 × ICC 8261 presented in this article).
