# Supplementary figures and images for: Genetic dissection of drought tolerance in chickpea (Cicer arietinum L.)
Source: Theor Appl Genet. 2013 Dec 11;127(2):445–62. doi: 10.1007/s00122-013-2230-6 (PMC3910274; doi:10.1007/s00122-013-2230-6)

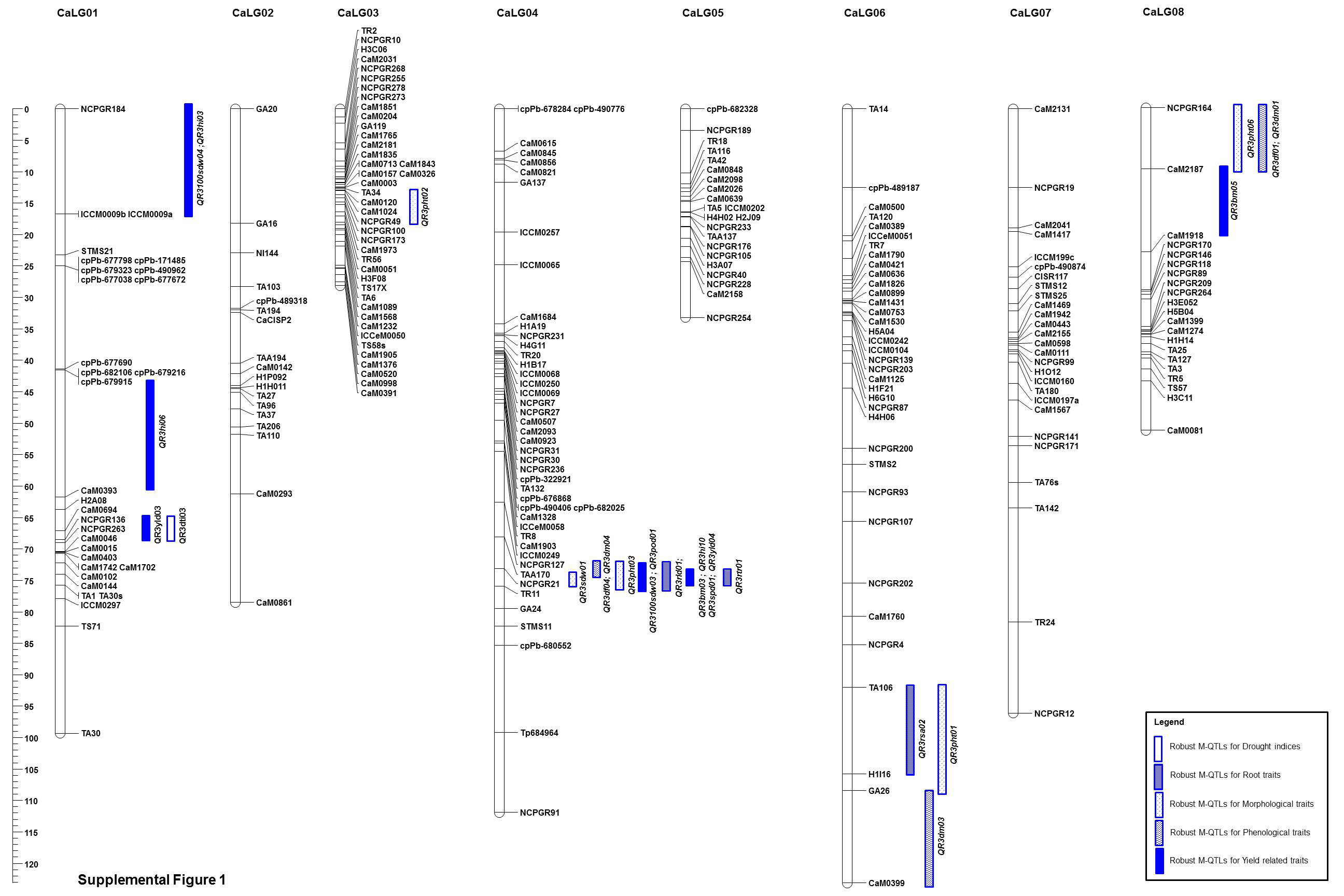

Supplement: Supplementary file 13 — ESM Figure S1: The intra-specific genetic map and QTL maps of chickpea constructed based on recombinant inbred line (RIL) mapping population ICC 4958 × ICC 1882 with 241 loci spanning 621.51 cM. The genetic distance in cM is represented on left hand side and the markers names are on the right hand side of the linkage group (TIFF 13128 kb) [file 122_2013_2230_MOESM13_ESM.tif]

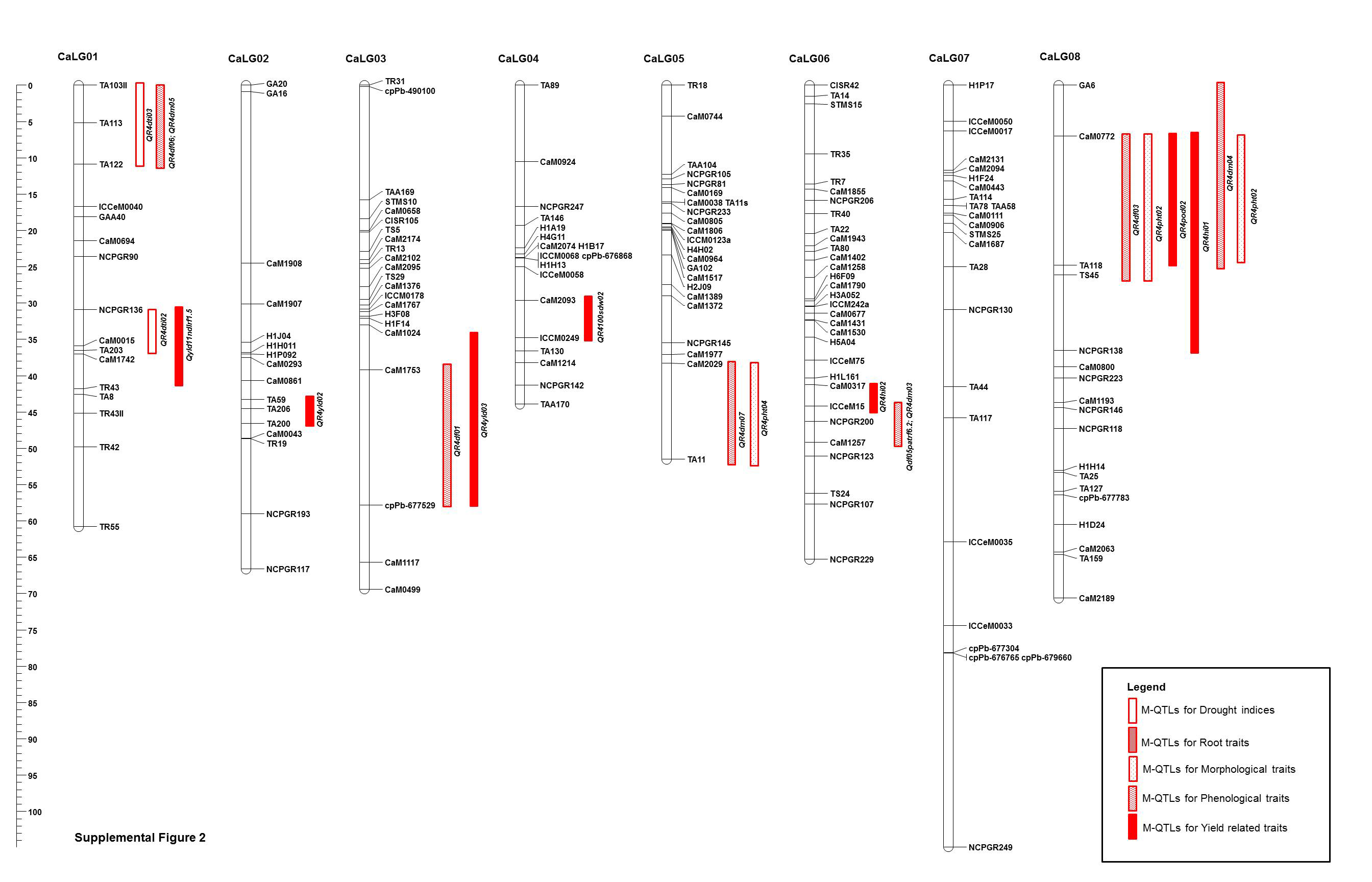

Supplement: Supplementary file 14 — ESM Figure S2: The intra-specific genetic map and QTL maps of chickpea constructed based on recombinant inbred line (RIL) mapping population ICC 283 × ICC 8261 with 168 loci spanning 533.06 cM. The genetic distance in cM is represented on left hand side and the markers names are on the right hand side of the linkage group (TIFF 13613 kb) [file 122_2013_2230_MOESM14_ESM.tif]
